# A New Strategy to Identify Naturally Presenting SLA-I Bound Peptides Derived from the O Serotype of Foot-and-Mouth Disease Virus, by Mild Acid Elution in a VP1 Stably Expressed PK15 Cell Line

**DOI:** 10.3390/ani15213097

**Published:** 2025-10-24

**Authors:** Yong-Yu Gao, Zong-Hui Zhang, Chen-Jun Sang, Yong Han, Yu-Die Cao, Yue Tang, Gui-Xue Hu, Zi-Bin Li, Feng-Shan Gao

**Affiliations:** 1 College of Animal Medicine, Jilin Agricultural University, Changchun 130118, China; gareth12138@163.com; 2 College of Life and Health, Dalian University, Dalian 116622, China; zzh_997@163.com (Z.-H.Z.); sangchenjun1212@163.com (C.-J.S.); hanyong2014boy@163.com (Y.H.); cyd4086@163.com (Y.-D.C.); 19818964152@163.com (Y.T.); lizibin@dlu.edu.cn (Z.-B.L.); 3 The Dalian Animal Virus Antigen Epitope Screening and Protein Engineering Drug Developing Key Laboratory, Dalian 116622, China

**Keywords:** foot-and-mouth disease virus, PK15, stable transfection, antigenic peptides, mild acid elution

## Abstract

**Simple Summary:**

The naturally presented antigenic peptides bound with swine leukocyte antigen class I (SLA-I) are crucial in developing multi-epitope vaccines in foot-and-mouth disease virus (FMDV). In this article, a transient expression plasmid, pEGFP-N1-VP1, was used to establish a VP1-stably expressed PK15 cell line. Using mild acid elution, 37 peptides that bound to SLA-I were separated and identified. These peptides will be used as candidates for a multi-epitope vaccine in FMDV.

**Abstract:**

Multi-epitopes of FMDV can be used to develop a novel vaccine. Determining how to screen naturally presenting epitope peptides derived from FMDV is crucial for advancing progress in this area. In this study, a transient expression plasmid named pEGFP-N1-VP1 was transfected into Porcine Kidney Epithelial cells 15 (PK15). The positive cells that stably expressed the *O-VP1* gene of FMDV were screened with gradient concentrations of G418 (Geneticin). The constructed pEGFP-N1-VP1/PK15 cell line was eluted by pH 3.3 phosphate buffer to isolate the eluted peptides, followed by desalting, liquid chromatography–tandem mass spectrometry (LC–MS/MS), a flow cytometric analysis of SLA-I expression, and an ELISA detection of SLA-I bound peptides. It was demonstrated that a PK15 cell line stably expressing the *VP1* gene was initially screened out at 500 μg/mL of G418, followed by culturing at 300 μg/mL. The O-VP1 expression was identified using an image analysis system, RT-PCR, and Western blot analysis. Thirty-seven peptides derived from O-VP1 were eluted from the constructed cell line. The flow cytometric analysis and ELISA detection results showed that the eluted peptides were associated with SLA-I and bound. This is the first known study to construct a cell line for screening naturally presenting antigenic peptides derived from the O serotype of FMDV.

## 1. Introduction

Foot-and-mouth disease (FMD) is a highly contagious and devastating infectious disease affecting livestock. It is caused by the FMD virus (FMDV), which belongs to the species *Aphthovirus vesiculae*, genus *Aphthovirus*, and family *Picornaviridae* [1]. Previously, FMD was widespread in Africa, South America, and Asia, significantly reducing livestock production and causing a severe economic impact on the livestock industry [2,3,4]. Currently, FMD continues to spread worldwide [5,6,7]. In early 2025, two unrelated strains of FMDV serotype O were discovered in Germany, Hungary, and Slovakia, indicating that FMD freedom in continental Europe was disrupted [8,9,10]. According to the serological tests, the FMDV can be divided into seven serotypes worldwide: A, O, C, Asia 1, and Southern African Territories (SAT) 1, SAT2, and SAT3 [11,12]. FMDV is made up of 60 particles, and each particle is mainly composed of four capsid proteins: VP1, VP2, VP3, and VP4. FMDV structural protein VP1 is the major antigenic fragment, which plays a crucial role in inducing immunity in animals [13,14,15]. Therefore, it is crucial to study the FMDV structural protein VP1 for vaccine development [16,17].

Currently, the method used to prevent FMDV infection in endemic areas involves injecting a chemically inactivated whole-virus vaccine into animals, which exhibits robust immunogenicity and provides protection. However, the inactivated vaccine is associated with drawbacks such as high production costs, the requirement for biosafety level 3 facilities in its production, potential biological safety risks during vaccine manufacture, and limited efficacy against antigenically variable strains [18,19,20]. In addition, this type of vaccine has a limitation in inducing cellular immunity [21,22]. These challenges underscore the need for alternative vaccine development. Nowadays, scientists have recognized a new approach that differs from traditional vaccines. This new method utilizes an antigenic immunodominant region to design an epitope vaccine, which comprises many epitopes composed of short peptides. The new vaccines do not carry any virus protein but can induce safe and effective immune protection [23]. Epitopes derived from viral proteins consist of B-cell epitopes, T helper (Th) epitopes, and cytotoxic T lymphocyte (CTL) epitopes [24]. Previous research on FMDV antigens has mainly focused on the study of B-cell and Th epitopes, which can mainly induce humoral immunity, but with less attention paid to CTL epitopes [25,26]. Recent studies have shown that CTL epitopes that bind to the peptide binding groove of the heavy chain of class I of the major histocompatibility complex (MHC) simultaneously noncovalently bind with light chain Beta 2 microglobulin (β_2_m), also play a key role against FMDV [24,27,28,29]. Crucially, these epitopes can induce CTLs to kill infected target cells and FMDV particles once they are presented on the surface of antigen-presenting cells (APCs), thereby inducing a cellular immune response in vivo [24,29,30]. Although humoral immunity is the primary defense against FMDV, for FMDV persistent infection and “carrier state” of animals, cellular immunity induced by CTL epitopes will be more effective in eliminating these states. Therefore, CTL epitopes along with B-cell and Th epitopes from FMDV might be used to develop a new type of FMDV vaccine that can not only supply safer protection to animals but also eradicate FMD completely in the future, by means of CTL epitopes to induce a cytotoxic cellular immunity to kill target cells along with the FMDV particles in animals. In addition, this type of vaccine, constituted by epitopes from different strains of FMDV, might be used to control these strains in the future.

In order to screen CTL epitopes from the VP1 protein of FMDV, many strategies were tried, including the construction of the covalently linked heavy chain and light chain of the swine MHC class I, i.e., SLA-I complex to screen antigenic peptides derived from FMDV [31,32]; and the renaturation of the heavy chain, light chain, and antigenic peptides derived from FMDV [27,33]. However, these strategies were all processed to screen peptides binding to SLA-I molecules in vitro; the screened peptides were not naturally presented by SLA-I molecules in vivo, which implies that the efficiency of screening CTL epitopes might be low. Previously, researchers had tried a mild acid-eluted peptide strategy applied to the surface of human or murine MHC class I complexed with naturally expressed tumor or other pathogen antigens in cell lines, which proved that the isolated antigenic peptides were naturally presented by MHC class I molecules and were functional in the developing vaccine [34,35,36]. However, to date, no swine cell lines have been used to screen and isolate antigenic peptides derived from FMDV, particularly those complexed with SLA-I and naturally expressed FMDV antigens in cell lines.

To establish a swine cell line for identifying naturally presented antigenic peptides from FMDV by SLA-I molecules, we transfected Porcine Kidney Epithelial 15 (PK15) cells with a primary transiently expressed plasmid pEGFP-N1-VP1. This process aimed to screen for a stably expressed *VP1* fusion gene cell line using G418 (Geneticin), followed by eluting the peptides presented by cells derived from the O serotype of FMDV. As a result, we successfully created the stably expressed pEGFP-N1-VP1/PK15 cell line, and 37 peptides from O-VP1 were eluted from it. Of these, 12 peptides showed relatively higher abundance values, and they were subsequently proven to bind to SLA-I in vitro, indicating that the eluted peptides should be presented by SLA-I.

## 2. Materials and Methods

### 2.1. Plasmids, Reagents, Cell Lines, and Primers

The plasmid pEGFP-N1-VP1 (a transient expression vector) was provided by Professor Shi-Jun Zheng from the China Agricultural University [37]. The expression plasmid SLA-1*04:01/pET-21a (+) was kindly given by Professor Chun Xia from the China Agricultural University. The expression plasmids SLA-2*04:02:01/pET-21a (+) and SLA-3*xd/pET-21a (+) were constructed by our group. The PK15 cell line was purchased from the China Veterinary Microbiology Culture Collection Center (CVCC, Beijing, China). Dulbecco’s Modified Eagle Medium (DMEM) and fetal bovine serum (FBS) were purchased from Gibco Company (Boston, MA, USA). A horseradish peroxidase conjugated goat anti-mouse Immunoglobulin G (IgG) monoclonal antibody, the SanPrep Column Plasmid Mini-Preps Kit, and the SanPrep Column DNA Gel Extraction Kit were purchased from Sangon Biotech Co., Ltd. (Shanghai, China). The reverse transcription kit, T4 DNA Ligase, and *Taq* polymerase were purchased from TaKaRa Biotechnology Co., Ltd. (Dalian, China). Lipofectamine^TM^ 2000 was purchased from Invitrogen Corporation (Waltham, CA, USA). G418 was purchased from Sigma-Aldrich (GoldBio, St. Louis, MO, USA). Mouse anti-Green Fluorescent Protein (GFP) monoclonal antibody was purchased from Beyotime (Shanghai, China). The monoclonal antibodies, including the heavy chain of SLA-I and the light chain β_2_m, were all produced and kept in our lab. Other antibodies are described and explained in Section 2. Acetonitrile (HPLC grade) was purchased from Kermel Company (Tianjin, China). Superstar Electrochemiluminescence (ECL) Plus Detection Reagent was purchased from Solarbio (Beijing, China). A pair of primers to detect *VP1* gene expression was taken from a previously reported [37], consisting of the forward primer VP1-F (5′-ACCACCTCCACAGGTGAGTCG-3′) and the reverse primer VP1-R (5′-CAAAAGCTGTTTCACAGGCG-3′). The primers were synthesized by Sangon Biotech Co., Ltd. (Shanghai, China).

### 2.2. Cell Culture

Cells were recovered from liquid nitrogen and cultivated at 37 °C in 5% CO_2_ for 12–18 h in a 10% FBS DMEM. The cell condition was monitored until they recovered their natural state. Then the medium was replaced by fresh DMEM containing 10% FBS. After the cells had grown and spread to the entire bottom of the flask, they were digested with 0.25% trypsin for approximately 5 min at 37 °C. Then, the digested cells were transferred into 24-well plates with 7–8 × 10^4^ cells per well. The cells were cultivated at 37 °C with a 5% CO_2_ for 24 h until the cell density reached about 70–80%.

### 2.3. G418 Lethal Concentration Curve Measurement

Approximately 4 × 10^5^ PK15 cells were transferred to 24-well plates and cultivated at 37 °C, 5% CO_2_. When the cell density reached approximately 70–80%, 500 μL of G418 was added to the 24-well plates at a series of concentrations: 0, 100, 200, 300, 400, 500, 600, 700, 800, 900, and 1000 μg/mL, diluted in DMEM containing 10% FBS. Each treatment was performed in triplicate wells at 37 °C and 5% CO_2_ in a culture incubator. Cells were observed daily. Every 48 h, the cells were treated with a fresh 10% FBS DMEM containing a gradient concentration of G418. The lowest G418 concentration that kills all cells within 7 days was identified as the final treatment dose for stable transfection of exogenous plasmids. Viable cells were counted by using an EVE Automatic cell counter (NanoEnTek, Seoul, Republic of Korea) as previously reported [38]. Then, the G418 lethal concentration curve for PK15 cells was drawn.

### 2.4. PK15 Cell Transfection Efficiency Assay

Approximately 4 × 10^5^ PK15 cells were transferred to 24-well plates and cultivated at 37 °C, 5% CO_2_. When the cell density reached 70–80% in each well, the normal DMEM containing 10% FBS was replaced by FBS-free OPti-DMEM before transfection. The PK15 cells were transfected with the lipofectamine^TM^ 2000/pEGFP-N1-VP1 complexes at ratios of 1:1–1:3, liposomes to plasmids. Transfection was performed in triplicate in each well, and the transfection efficiency, represented by the percentage of EGFP-positive cells, was determined by flow cytometry. Simultaneously, a negative control for PK15 cells, consisting of no liposomes and plasmids, was set up and detected as described above.

### 2.5. PK15 Cell Transfection and G418 Screening

For these experiments, 4 × 10^5^ PK15 cells were transferred to 24-well plates and cultivated at 37 °C, 5% CO_2_. When the density of the transfected PK15 cells reached 70–80% of each well, lipofectamine^TM^ 2000/pEGFP-N1-VP1 was transfected into the cells at a ratio of 1:1. After transfection for 24 h, the transfected cells were washed twice by Phosphate-Buffered Saline (PBS), and the medium was refreshed by DMEM containing 10% FBS and G418 (500 μg/mL) once after 48 h. After 7 days, the cell masses emitting clear green fluorescence in the well were marked, and 2 μL of 0.25% trypsin was gently pipetted onto them using a micro-pipette. The cells were then transferred to new 96-well plates to cultivate. The cells in the 96-well plates were observed using fluorescence inverted microscopy until they were clearly visible and growing in number. The cells in the 96-well plates were then transferred into 24-well plates and maintained with medium containing 10% FBS and G418 (500 μg/mL). After the cells had grown to fully cover the well, they were transferred to 6-well plates and cultivated in medium containing 10% FBS and G418 (500 μg/mL), and then to a 6 cm culture dish. Finally, the cells were transferred to a 10 cm culture dish for further cultivation and passage, and then stored in a −80 °C refrigerator. At various time points after transfection–3, 6, 12, 24, 48, 72, 168, 336, and 480 h–some cells were used to detect and evaluate the effects of transfection and screening on the transfected PK15 cells. In brief, the transfected cells were fixed with 4% paraformaldehyde, incubated for 30 min at room temperature, and then washed three times with PBS. Then, Triton X-100 was added to the fixed cells to a final concentration of 0.2% to permeate the cellular membrane for 10 min, followed by three washes with PBS. Then, 4′,6-diamidino-2-phenylindole (DAPI) was added to the cells to a final concentration of 1 μg/mL to dye the cell nucleus for 10 min, followed by washing with PBS three times. The cells were detected using an ImageXpress Micro XLS Widefield High-Content Analysis System (Molecular Devices, Sunnyvale, CA, USA) under a 20× plan objective lens and photographed by using the DAPI and Fluorescein Isothiocyanate (FITC) channels. The data were analyzed using Thermo Scientific HCS Studio 2.0 (Waltham, MA, USA).

### 2.6. Identification of the Stable Expression of the VP1 Gene in pEGFP-N1-VP1/PK15 Cells

To confirm whether the target plasmid was successfully transfected into PK15 cells and to identify the transcription of the *VP1* gene in cells, total RNA was extracted from the transfected cells using the TRIzol Reagent kit (Invitrogen Inc., Carlsbad, MA, USA) according to the previously reported methods [39]. The RNA samples were stored at −80 °C, ready for use. The extracted total RNA was reverse-transcribed into cDNA using avian myeloblastosis virus (AMV) reverse transcriptase (TaKaRa, Dalian, China) and oligo (dT) primers according to the manufacturer’s recommendations. A template of 1 μg of cDNA was used to amplify the *VP1* gene with the primers VP1-F and VP1-R. The PCR reaction was conducted as follows: 94 °C for 5 min for pre-denaturation, followed by 94 °C for 30 s, 56 °C for 45 s, and 72 °C for 1 min, for 30 cycles; finally, 72 °C was maintained for 10 min. The PCR product was stored at −20 °C for subsequent gene cloning. The PCR product was recovered by a SanPrep Column DNA Gel Extraction Kit (Sangon Biotech Co., Ltd., Shanghai, China), cloned into the pMD 18-T vector, and then sequenced.

For the identification of VP1 protein expression in pEGFP-N1-VP1/PK15 cells, 2 × 10^6^ stably transfected cells were collected to isolate intracellular proteins by the Radio Immunoprecipitation Assay (RIPA) lysis buffer. Then, the protein concentration was determined with the Bicinchoninic Acid (BCA) method [40]. Next, 20 μg of denatured protein samples were determined by SDS-PAGE and then transferred to a 0.45 μm Polyvinylidene Divinylidene (PVDF) membrane. The membrane was blocked for 1 h at room temperature with PBS containing 5% skim milk, and incubated with a VP1 antibody (1:1000, mouse origin, kindly offered by Professor Shi-Jun Zheng from the China Agricultural University, Beijing, China), and a referenced Glyceraldehyde-3-phosphate Dehydrogenase (GAPDH) mouse monoclonal antibody (Proteintech Group, Inc., Chicago, IL, USA) at 4 °C. The samples were washed three times for 10 min each time with PBS containing 0.3% Tween 20. The membrane was incubated for 1 h at room temperature with horseradish peroxidase (HRP) labeled with goat anti-mouse IgG monoclonal antibody (1:5000), and then visualized using Superstar ECL Plus Detection Reagent (Solarbio, Beijing, China). Immunoreactive bands were quantified densitometrically to analyze the relative expression of VP1 using ImageJ software (1.53k; NIH, Bethesda, MD, USA), with GAPDH serving as the loading control.

### 2.7. Detection of the Peptides Eluted with Mild Weak Acids from the Surface of pEGFP-N1-VP1/PK15 Cells by LC–MS/MS

In order to elute and detect peptides, the pEGFP-N1-VP1/PK15 cells were cultivated to the logarithmic phase in DMEM containing 10% FBS and 300 μg/mL G418 at 37 °C and 5% CO_2_. Then 1 × 10^8^ cells were collected, washed twice with PBS, and centrifuged at 1500 rpm for 5 min at room temperature. The cells were then eluted with phosphate buffer (0.131 mol/L citric acid, 0.066 mol/L Na_2_HPO4, pH 3.3) for 30 s at room temperature, as described by Storkus et al. [36], with modifications tailored to our system. The elution mixture was centrifuged at 1500 rpm for 5 min to remove cells. The elution sample was centrifuged at 4500× *g* for 5 min using an Amicon Ultra-15 Protein Centrifugal Filter (3000) to remove large fragments of proteins or other large molecules. The filtrates were desalted using Stage-tip C18 (Empore^TM^ Octadecyl C18, 47 mm Extraction Disks, MA, USA) according to slightly modified previously reported methods [41]. First, 100 μL of acetonitrile was added to the membrane of the Stage-tip C18 until the acetonitrile fully flowed through the membrane. The C18 column was balanced by 100 μL of 0.1% Trifluoroacetic Acid (TFA)/H_2_O and then centrifuged at 500× *g* until the solution fully flowed through the membrane. The balance process was repeated three times. The column was transferred to a new 2 mL Eppendorf (EP) tube, and then the peptide samples were loaded onto the column membrane and incubated for 5 min at room temperature. This was followed by centrifugation at 500× *g* until the samples had all flowed through. The filtrate was reloaded onto the membrane to repeat the filtration. The column was washed three times with 100 μL of 0.1% TFA/H_2_O and centrifuged three times at 500× *g*, as above. The column was transferred to a new 2 mL Eppendorf tube. Then, 100 μL of elution buffer containing 0.1% TFA and 50% acetonitrile was used to elute the peptide samples absorbed into the column membrane, followed by centrifugation at 400× *g* until the buffer had fully flowed through. The elution was repeated twice. The two eluates were combined and collected in a 500 μL Eppendorf tube for lyophilization in a SpeedVac concentrator (Hunan Hercy Instrument Co., Ltd., Changsha, China), completing the drying process.

The lyophilized peptides were dissolved in 20 μL of a 0.1% formic acid/water solution. Then, 4 μL of samples were injected into a C18 (3 µm, 100 Å, 75 µm × 15 cm), and separated through a 90 min chromatographic gradient using high performance liquid chromatograph (HPLC, Thermo Scientific^TM^ Easy nLC 1200, Waltham, MA, USA), with a balance between mobile A phase of 0.1% formic acid in water and mobile B phase of 0.1% formic acid in 80% acetonitrile/H_2_O at a flow velocity of 350 nL/min.

Mass spectra data were detected using an Orbitrap Fusion Lumos ultra-high resolution mass spectrometer (Thermo Scientific, Waltham, MA, USA) with the following parameters: spray voltage, 2.0 kV; capillary temperature, 320 °C; RF Lens, 40. The resolution was set at 120,000 at *m*/*z* 200 for primary MS data and 30,000 at *m*/*z* 200 for MS/MS data. The parent ion scanning range was from 350 to 1550 *m*/*z*, while the daughter ion *m*/*z* scanning started at 110 *m*/*z*. The ion screening window was set at 1.6 *m*/*z*. The fragmentation mode was set at high-energy collision dissociation (HCD). The energy was selected as normalized collision energy (NCE) 32. The data-dependent MS/MS was first set at the top 20 and then analyzed artificially. The dynamic exclusion time was set at 60 s. The data were analyzed by Proteome Discoverer 2.1 (Thermo Fisher, MA, USA).

### 2.8. Detection of the Eluted Peptides Associated to SLA Class I-Presenting

To determine whether the eluted peptides were associated with SLA-I binding and presentation, a flow cytometric analysis of SLA-I expression was conducted, as previously reported [42] and modified. In brief, the monoclonal antibody of the heavy chain of SLA-I, light chain β_2_m (both produced and kept in our laboratory) and SLA-I complex antibody (produced in Hubei Qiangyao Biological Technology Co., Ltd., (Ezhou, China) and kept in our lab) were used to detect the expression of SLA-I on the surface of cells before and after elution with phosphate buffer. The acid-treated cells were immediately washed with PBS at pH 7.2 and then re-cultured in RPMI 1640 containing 10% FBS. Cells at a concentration of 4 × 10^5^ were collected and seeded in a 1.5 mL centrifuge tube. The cells were then washed with PBS buffer and blocked with 3% BSA for 15 min. The processed cells were incubated with one of three monoclonal antibodies against the heavy chain, β_2_m, and SLA-I complex, respectively, for one hour at 4 °C. Then, APC-labeled Rat anti-Mouse IgG1 clone (Becton Dickinson, San Jose, CA, USA) was used as a secondary antibody and incubated with the above-processed cells for one hour at 4 °C. A negative control was established using the primary PK15 cells processed as described above. All treated cells were detected using a FACS-Calibur (Becton Dickinson Immunocytometery System, San Jose, CA, USA) and analyzed with FlowJo software (Becton Dickinson Immunocytometry Systems, version 10, Ashland, OR, USA).

Meanwhile, in order to identify whether the eluted peptides were bound to SLA-I molecules, some representative eluted peptides, along with a positive peptide As64 (ALLRSATYY) [33] and a control random peptide CN (DPSPGQSCN), were synthesized linearly in GenScript Corporation (Suzhou, China). A peptide binding affinity assay was adapted from published micro-scale refold-ELISA-based methods to detect the binding affinity of identified peptides with the SLA-I complex, with moderate modifications [43,44]. In brief, 1 μmol/L heavy chain (HC) of SLA-I and 1.5 μmol/L pre-refolded β_2_m were refolded in 0.33 mmol/L Tris-Maleate and 0.5% Lutrol-F68 in the presence of 70 μmol/L peptide, prediluted to 2 mmol/L working stocks in 100 mmol/L Tris-HCL, pH 8.0. Micro-refolds were incubated at 18 °C for 48 h before the relative capacity of each peptide to support stable SLA-I-peptide-β_2_m complex formation was quantified by sandwich ELISA. After diluting the HC antibody (produced in Hubei Qiangyao Biological Technology Co., Ltd., and stored in our lab) to 10 μg/mL in 2% BSA, 50 μL per well was added to the ELISA plate, and coated overnight. The plates were washed 3 times with 0.05% Tween 20 + 2% BSA, then blocked with 5% BSA at room temperature for 1 h. Subsequently, the plate was washed 3 times with 0.05% Tween 20 + 2% BSA. The SLA-I-Peptide-β_2_m renaturation product was diluted to 1:100, and 100 μL was added to each well of the ELISA plate. The solution was then incubated at 4 °C for 2 h; 0.05% Tween was used to wash the plate 5 times with 20 + 2% BSA, 100 μL per well of HRP-labeled rabbit-anti swine β_2_m polyclonal antibody (produced in Hubei Qiangyao Biological Technology Co., Ltd., and kept in our lab), to achieve a final concentration of 0.2 μg/mL, and incubated for 1 h at 4 °C; the plate was washed 5 times with 0.05% Tween 20 + 2% BSA. HRP-labeled goat-anti rabbit IgG (abcam) was diluted with 2% BSA to 0.02 μg/mL and incubated at room temperature for 1 h. Tetramethyl benzidine substrate and STOP solution were used to progress and terminate reactions, respectively, before obtaining absorbance readings at 450 nm on a Bio-Rad 680 Microplate Reader (Hercules, SA, USA). Each detection was set as a triplicate well.

### 2.9. Statistical Analysis

All values were expressed as mean ± SD. Experimental results are presented as mean ± standard error of mean (SEM) (minimum 3 repeats). Analyses were conducted in GraphPad Prism 8 (GraphPad Software, San Diego, CA, USA). One-way analysis of variance (ANOVA; repeated measures test) was used to determine the significance of differences among the groups. Statistical significance was set at *p* < 0.05.

## 3. Results

### 3.1. G418 Lethal Concentration Curve

The PK15 cell lethal assay was performed over seven days using a series of G418 concentrations. A curve illustrating the relationship between the mean number of viable cells and G418 concentration was plotted, as shown in Supplementary Figure S1. It demonstrates that when the G418 concentration reaches 500 μg/mL, all cells are killed. Therefore, the effective G418 concentration for screening stable transfectants is determined to be 500 μg/mL.

### 3.2. Detection of the Transfection Efficiency of PK15 Cells by Flow Cytometry for Different Liposome Complex Ratios

**Figure 1:**
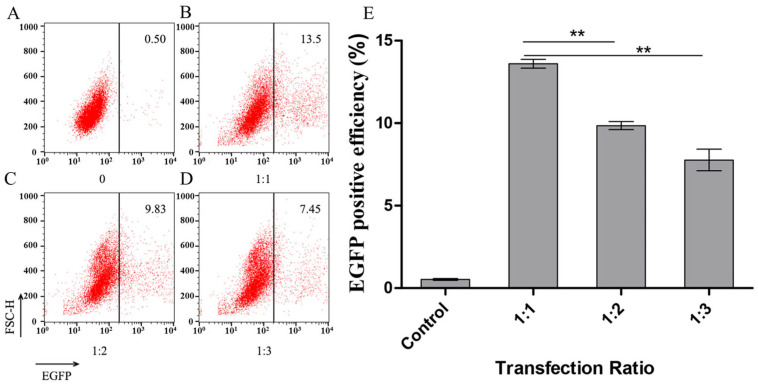
Detection of the transfection efficiency of PK15 cells by flow cytometry. (**A**) A representative detection of the control PK15 cells with no transfectant is marked as 0 under the *X*-axis of the figure. 0.50 is represented as a background value for the detection. (**B**–**D**) Representative detections of the transfection efficiency of PK15 cells at (**B**) 1:1, (**C**) 1:2, and (**D**) 1:3 ratios of liposomes to plasmids. The transfection efficiency was 13.5, 9.83 and 7.45 percent, respectively. (**E**) Analysis of the transfection efficiency of PK15 cells at different ratios of liposomes to plasmids by statistics. ** *p* < 0.01.

The transfection efficiency of the transfected cell line was detected by flow cytometry after 6 h for different ratios of liposome to plasmid; for 1:1, 1:2, and 1:3. When the ratio was 1:1, 1:2, and 1:3, the transfection efficiency of PK15 cells represented by expressed EGFP positive efficiency was 13.5, 9.83 and 7.45 percent, respectively. It was demonstrated that the transfection efficiency at a 1:1 ratio was significantly higher than at the other ratios, despite a 0.5% background value in the negative control, as shown in Figure 1.

### 3.3. Detection of the VP1-Fused Green Fluorescent Protein Expression in Stably Transfected Cells

**Figure 2:**
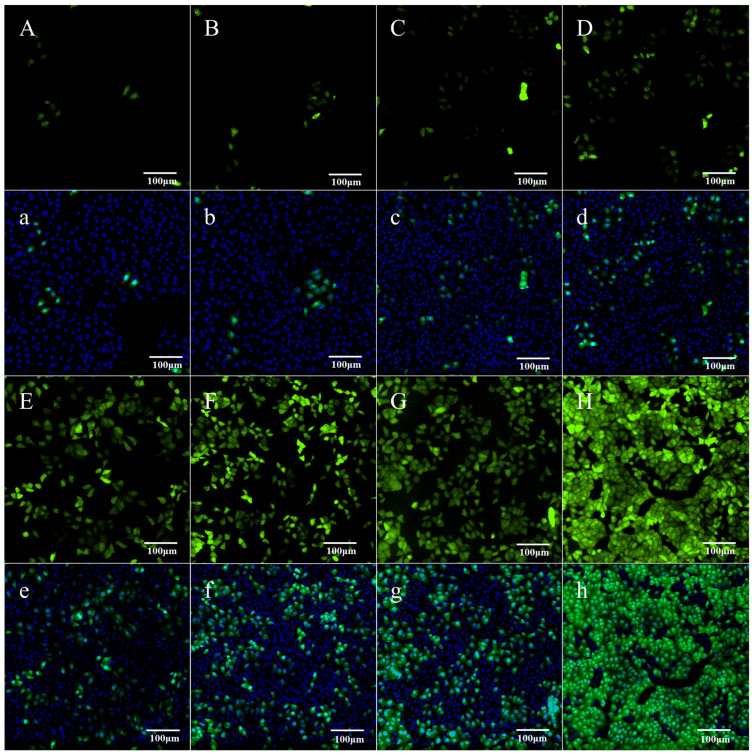
Detection of transfection results for pEGFP-N1-VP1 vectors at different times after transfection. (**A**–**G**) Detection of the expression of fluorescence associated VP1 fused to EGFP protein, which emits light green for transfected PK15 cells when measured by an ImageXpress Micro XLS Widefield High-Content Analysis System. The measurement times are 3, 6, 12, 24, 48, 72, and 168 h after transfection, respectively. (**a**–**g**) Detection of the expression of both fluorescence-associated proteins and the nucleus stained with 4′,6-diamidino-2-phenylindole for the systems corresponding to A–H. The cells with only blue nucleus staining are negative cells for VP1 fused to EGFP expression, while the cells with dark green cytoplasm and cyan nucleus stains are positive cells for VP1 fused to EGFP expression. (**H**) An analogous measurement to (**A**–**G**) after increasing cultivation from a cell mass emitting fluorescence. This measurement was taken 336 h after transfection. (**h**) An analogous measurement to (**a**–**g**) for the same system as (**H**).

**Figure 3:**
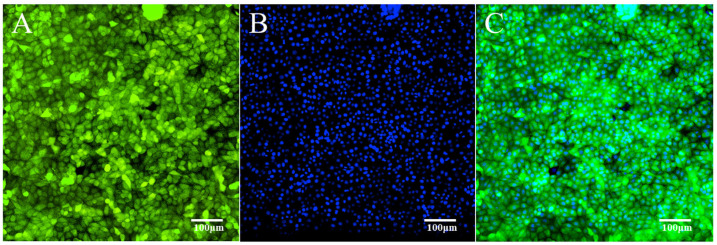
(**A**) Detection of the expression of VP1 fused to EGFP protein (green) in the stably transfected pEGFP-N1-VP1/PK15 cell line at 480 h after transfection. (**B**) Detection of the cell nuclei stained with DAPI in light blue for the same system. (**C**) A composite image of (**A**,**B**). The cells with nuclei stained only blue are negative for EGFP-VP1 expression, while the cells with cytoplasm stained dark green and nuclei stained cyan are positive for VP1 fused to EGFP expression.

By using a 1:1 ratio of liposomes to plasmids, the pEGFP-N1-VP1 was successfully transfected into PK15 cells and detected at different times by an ImageXpress Micro XLS Widefield High-Content Analysis System (Molecular Devices, Sunnyvale, CA, USA), as shown in Figure 2. Screening under 500 μg/mL of G418 has revealed that transfected PK15 cells with green fluorescence can be detected after 3 h; however, they do not grow by 6 h, indicating that many transiently expressed cells were killed during this time. From 12 h onward, the number of transfected cells with green fluorescence increased (Figure 2A–F,a–f). At 7 days (168 h), a few cell masses displaying clear green fluorescence formed (Figure 2G,g). Then, the marked cell masses were transferred to 96-well plates and cultivated with DMEM containing 500 μg/mL of G418. After 10 days, wells that still displayed stable green fluorescence were selected as positive cell wells. The positive cells were progressively cultivated from 96-well to 24-well plates under the same conditions as above, followed by 6-well plates, a 6 cm culture dish, and a 10 cm culture dish, in sequence. Figure 2 shows that at 14 days (336 h), more than 90% of cells were fluorescent (Figure 2H,h). Through continued screening and cultivation, nearly 100% of the transfected cells were fluorescent at 20 days (480 h) after transfection, as shown in Figure 3, indicating that the cells stably express the VP1-fused EGFP protein.

### 3.4. Detection of the Expression of the VP1 Gene in pEGFP-N1-VP1/PK15 Cells

**Figure 4:**
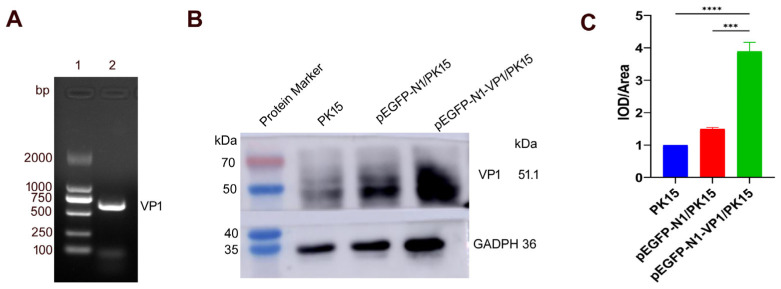
Identification of the stable expression of VP1 in the pEGFP-N1-VP1/PK15 cell line. (**A**) Detection of *VP1* transcription from the stably transfected pEGFP-N1-VP1/PK15 cell line by RT-PCR. Lane 1, DNA 2000 Maker; lane 2, the amplified *VP1* fragment from stably transfected cells. (**B**) Detection of VP1 expression in the stably transfected pEGFP-N1-VP1/PK15 cell line by a Western blot assay. The reference protein is Glyceraldehyde-3-phosphate Dehydrogenase (GAPDH), with a molecular weight of about 36 kDa, while the fusion VP1-EGFP protein has a molecular weight of about 51.1 kDa. The control PK15 cells and the transfected pEGFP-N1/PK15 cells did not express the protein of interest. (**C**) Analysis of the relative expression of VP1 in contrast to PK15 and pEGFP-N1-VP1/PK15 controls, respectively. **** *p* < 0.0001; *** *p* < 0.001.

To identify whether *VP1* was stably expressed in the mRNA of the pEGFP-N1-VP1/PK15 cells, RT-PCR was performed to detect the *VP1* transcription product. The results of this, in Figure 4, show that a specific amplified fragment was about 600 bp, which is consistent with the theoretical value of 639 for the length of the stably transcribed *VP1* in pEGFP-N1-VP1/PK15 cells (Figure 4A). The PCR product was recovered using a SanPrep Column DNA Gel Extraction Kit (Sangon Biotech Co., Ltd., Shanghai, China) and ligated into the pMD 18-T vector, which was then transferred to TOP 10 competent cells. Positive clones were selected for sequencing. The sequencing results show that the sequence of the *VP1* clone was completely consistent with that of *VP1* in the pEGFP-N1-VP1 plasmid (Supplementary Figure S2). Western blot results (Figure 4B) demonstrate that the VP1 fusion protein was stably expressed in the transfected PK15 cell line, as confirmed by the anti-VP1 monoclonal antibody (mAb). The control PK15 cells and the transfected pEGFP-N1/PK15 did not express the protein. The fusion protein had a molecular weight consistent with the EGFP protein, which is 28.0 kDa, and the VP1 protein, which is 23.1 kDa. Therefore, the theoretical value of the molecular weight of the VP1 fusion protein should be about 51.1 kDa, which is consistent with the measurements shown in Figure 4B. By the qualification of the relative expression of VP1, it was shown that the expression of VP1 fusion protein in pEGFP-N1-VP1/PK15 cells was quite significantly higher than that in PK15 and pEGFP-N1/PK15 controls (*p* < 0.001), respectively, indicating that the VP1 fusion protein was expressed truly (Figure 4C).

### 3.5. Detection of the Peptides Eluted with Mild Weak Acids by LC–MS/MS

The eluted peptide sample was detected using a high-performance liquid chromatography and an Orbitrap Fusion Lumos ultra-high-resolution mass spectrometer. The primary data was analyzed by Proteome Discoverer 2.1. The peptides were searched for in a self-constructed O-VP1.fasta database and screened using a fixed-value Peptide-Spectral Match (PSM) validator. A medium screening standard was used. After analysis, at least 37 peptides with 8–11 amino acids, derived from O-VP1.fasta were identified, as shown in Table 1. They just fit the antigen-binding groove of MHC class I molecules [45]. Most of the peptides were modified after being presented, such as through oxidation or deamination. Among them, the first 12 peptides displayed a relatively higher abundance value (XCorr Sequest High Throughput value over 1). The mass spectrogram of the first 12 peptides is shown in Supplementary Figure S3.

### 3.6. Detection of the Eluted Peptides Associated with SLA-I Binding and Presenting

**Figure 5:**
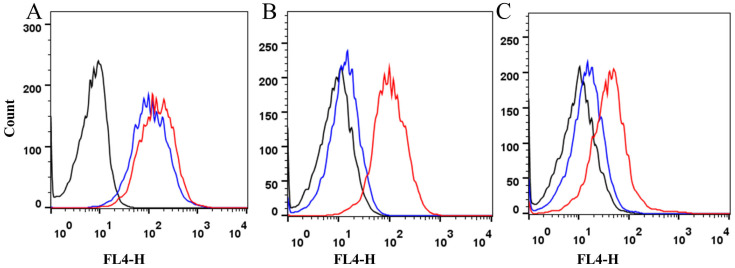
Flow cytometric analysis of SLA-I expression by pEGFP-N1-VP1/PK15 cells before and after acid elution treatment. The SLA-I expression on the surface of different cells was detected by (**A**) heavy chain monoclonal antibody, (**B**) light chain β_2_m monoclonal antibody before and after acid elution treatment, and (**C**) SLA-I antibody. The black line indicates the negative control cell of primary PK15; the red line indicates SLA-I expression before acid elution treatment; the blue line indicates SLA-I expression after acid elution treatment.

As shown in Figure 5, flow cytometric analysis showed that the SLA-I expression on the cell surface of the pEGFP-N1-VP1/PK15 was greatly decreased after acid elution treatment, detected by the light chain β_2_m monoclonal antibody and the SLA-I complex antibody, which indicated that β_2_m, along with eluted peptides, was lost after acid elution treatment. However, it seems that the heavy chain expression on the pEGFP-N1-VP1/PK15 changed little after acid elution treatment, which indicated that the heavy chain of the SLA-I could still remain associated with the cell surface on the pEGFP-N1-VP1/PK15 cells.

**Figure 6:**
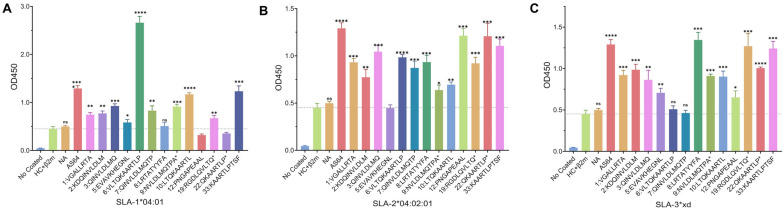
Peptides’ binding affinity with SLA-I was detected by ELISA. (**A**) Peptides binding affinity with SLA-1*04:01, (**B**) Peptides binding affinity with SLA-2*04:02:01, (**C**) Peptides binding affinity with SLA-3*xd. No coating represented a blank control, which consisted of no coating with HC antibody and no antigen added to the well, although HRP-labeled β_2_m antibody and HRP-labeled goat anti-mouse IgG were added. HC + β_2_m represented a negative control. NA represented a negative peptide. As64 represented a positive control. Data for three biological repeats with average absorbance ± SEM are reported. A significant eluted peptide was identified by comparing it with the negative control at a threshold of *p* < 0.05. **** *p* < 0.0001; *** *p* < 0.001; ** *p* < 0.01; * *p* < 0.05; ns, non-significant.

A micro-refolding and sandwich ELISA-based approach enabled relative quantification of peptide binding affinity for SLA-1*04:01, SLA-2*04:02:01 (completely matched to SLA-2*PK15) and SLA-3*xd (completely matched to SLA-3*PK15). Thirteen eluted peptides, including most of the first 12 peptides (No.4 and No.11 eluted peptides were not used because of the same linear amino acids as No.3 and No.7, respectively), and three after the 12th peptides, No.19, 22 and 33, were detected. In addition, one peptide As64 [27,29] and one random peptide NA (DPSPGQSCN) were used as positive and negative controls, respectively, while the refolded formation of a heavy chain (HC) of SLA-I and light chain of β_2_m was used as a negative control. As shown in Figure 6A, for the SLA-1*04:01 molecule, compared to the negative control, except for No.8, 12, and 22, all of the other eluted peptides were significantly more strongly bound with SLA-1*04:01, just like the positive peptide As64, while the control random peptide CN was not. Among them, the No.6 peptide showed the strongest binding affinity with SLA-1*04:01, even stronger than the positive peptide As64. Moreover, two other peptides, namely No. 10 and 33, which showed a comparative binding affinity with As64 to SLA-1*04:01, were previously cloned from PK15 cells [46]. For SLA-2*04:02:01, which were completely matched to SLA-2 derived from PK15, shown in Figure 6B, most of the eluted peptides showed significant binding against the negative control except for No.5. However, no peptide showed a stronger binding affinity with SLA-2:04:02:01 than As64, although including three comparative peptides No.12, 22 and 33 with As64 in binding affinity to SLA-2:04:02:01. For SLA-3*xd molecules, completely matched to SLA-3 from PK15, there were two peptides, notably No.6 and 7, showing negative binding affinity with SLA-3*xd, and other peptides showed significantly binding affinity with SLA-3*xd, as shown in Figure 6C. Just similar to SLA-2:04:02:01, there are three peptides, specifically No.8, 19 and 33, showing comparative binding affinity to SLA-3*xd.

## 4. Discussion

Nowadays, multi-epitope vaccines are one of the alternatives to traditionally inactivated FMDV vaccines due to their safety and efficiency in resisting different serotypes of the virus [47,48]. To design multi-epitope FMDV vaccines, identifying new epitopes, especially CTL epitopes, should be the key task. In fact, some researchers have begun to study the CTL epitopes derived from FMDV that can induce CD8^+^ T lymphocyte-dominated cellular immunity [31,49]. However, to date, these epitopes have been mostly designed using the conventional method of computer prediction tools [24,45] or by in vitro renaturation complexed with the heavy chain of SLA-I and the light chain of swine β_2_m [27,41,46], rather than the naturally presented peptides. Up to now, there are still no reports that a specific cell line was used to isolate the naturally presented SLA-I-bound peptides derived from FMDV.

In this study, we established a VP1-transfected PK15 cell line that stably expresses the *VP1* gene of the O serotype of FMDV. The *VP1* gene fused with the EGFP gene was expressed in cells, as detected by RT-PCR and Western blot analysis. Since the *VP1* gene was fused to EGFP, the cells that stably expressed the *VP1* gene should have displayed green fluorescence, as detected by an ImageXpress Micro XLS Widefield High-Content Analysis System. The results indicate that the VP1 proteins were expressed in the cytoplasm of cells as endogenous antigens, as shown in Figure 3 and Figure 4. According to the mechanism of processing and presenting the endogenous antigen of cells, the endogenously expressed VP1 proteins should be processed and degraded into peptides after being digested through proteasomes [50,51,52]. Some of the peptides were transported to and bound to the heavy chain of MHC class I molecules, which was ligated non-covalently to the light chain (β_2_m) to form a complex. The complex was refolded in the Golgi apparatus and then presented onto the surface of the cellular membrane to bind CD8^+^ T lymphocytes and induce cellular immunity [53]. If the peptides bound to the MHC class I molecules were eluted and extracted, natural CTL epitopes could be identified [54,55]. In this study, the primary objective was to establish a PK15 cell line that could stably express VP1 using a transient expression vector, pEGFP-N1-VP1, allowing the VP1 protein to be continuously processed and presented by the cells. During the establishment, we found that the concentration of G418 and the ratio of liposomes to plasmids were crucial points in screening PK15 cell transfectants to stably express VP1. After a long selection process, stably expressed VP1 fused EGFP transfected PK15 cells, obtained with a concentration of G418 at 500 μg/mL and a 1:1 ratio of liposomes to plasmids, were screened, isolated, and gradually increased in number through cultivation. However, while cultivating the complete positive cells, the concentration of G418 was adjusted to 300 μg/mL to ensure that the *VP1* gene fused with the EGFP gene was stably expressed in PK15 cells.

To determine whether the *VP1* gene fused with the EGFP gene was stably expressed, we performed RT-PCR and Western blot assays on the cells. These results successfully demonstrate that *VP1* fused with the EGFP gene is expressed at both the nucleic acid and protein levels. The above results indicate that we successfully constructed a cell line that stably expresses the O serotype of the *VP1* gene, which can then be used for peptide elution. However, to date, most reports on acid-eluted antigenic peptides have been conducted in human or murine natural cell lines isolated from tissues associated with tumors or direct viral infection [34,35,56]. As for swine, there is still no report associated with the identification of the naturally presenting peptides by acid-elution. Because FMDV pathogen experiments require high-grade biosafety, it is not a feasible strategy for most labs or groups to obtain cells that can directly express the VP1 protein from FMDV-challenged tissues. Therefore, constructing a cell line that stably expresses the VP1 protein should be the key strategy to solve this problem. In this research, we transfected the transiently expressed plasmid pEGFP-N1-VP1 into PK15 cells using a G418 gradient concentration screening method and a gradient-enlarged culture. A cell line that stably expresses the VP1 protein has been successfully constructed in our lab. Then, using the mild acid elution method, we identified 37 eluted peptides, each with 8–11 amino acids, from the surface of the constructed pEGFP-N1-VP1/PK15 cell line by liquid chromatography–tandem mass spectrometry (LC–MS/MS). Among them, the first 12 peptides with XCorr Sequest HT values (abundance values) over 1 are the top higher-reliability antigenic peptides, as shown in Table 1 and Supplementary Figure S3. The peptide binding groove of SLA-I, similar to the other MHC class I, can mostly accommodate 8–11 amino acids, which just matches the length of the eluted peptides in this assay [45,57]. The flow cytometric analysis further confirmed that the eluted peptides should be associated with and presented by SLA-I molecules. This is because the peptides, along with the light chain of β_2_m, bound to the heavy chain of SLA-I through hydrogen bonds, were eluted and consequently lost. This finding aligns with previous reports on other MHC class I detection methods [32,37]. However, because the heavy chain of SLA-I can be embedded on the surface of cells through transmembrane and intracellular regions, just like other MHC class I [53], the expression of the heavy chain of class I on the cell surface decreased slightly, as shown in Figure 5. This differs from Santin et al., who reported that the expression of the heavy chain of MHC I in tumor cells increases after acid treatment [42]. It may also be due to the varying levels of MHC I expression in different cells. There are three constitutively expressed classical and polymorphic SLA-I genes in the genome, namely SLA-1, SLA-2 and SLA-3. Among them, SLA-1 and SLA-2 are more polymorphic than SLA-3, which means that SLA-1 and SLA-2 are used more frequently in APCs [58]. Therefore, in order to prove that the eluted peptides were SLA-I bound, we adopted a micro-scale refold-ELISA-based method to detect the binding affinity of eluted peptides with SLA-1*04:01, SLA-2*04:02:01, and SLA-3*xd, all of which were isolated from PK15 cells. The ELISA results indicated that all of the eluted peptides were significantly bound with at least one of three SLA-I molecules compared to the negative control, while the control random peptide CN showed non-significantly bound with SLA-I, as shown in Figure 6, although some of the peptides did not display a significant peptide-binding affinity with one of SLA-1*04:01, or SLA-2*04:02:01, or SLA-3*xd. Therefore, it can be deduced that all the eluted peptides were SLA-I bound, although different peptides showed different peptide-binding preferences with different SLA-I molecules. For example, the No. 8 peptide did not display a significant peptide-binding affinity with SLA-1*04:01, but showed extremely significant peptide-binding affinity with SLA-1*04:02:01 and SLA-3*xd. Other peptides, such as No.12, 22, 5, 6 and 7 had a similar phenomenon to peptide No.8. Based on these data, it could be deduced that the SLA-1, SLA-2 and SLA-3 in PK15 cells can cooperatively present peptides. However, it seems that SLA-1*04:01 and SLA-2*04:02:01 play a more crucial role in peptide-presenting than that of SLA-3*xd, because SLA-1*04:01 can bind a dominant peptide No.6 with the highest affinity, while SLA-2*04:02:01 covers a more comprehensive range of peptide-binding than SLA-3*xd. It should be noted that the order of each peptide-binding affinity was not completely consistent with that of the abundance value shown in Table 1, which might be attributed to the different environment for peptides in vivo or in vitro. However, we proved that the eluted peptides were SLA-I-associated and bound. These results indicate that the eluted peptides derived from the VP1 protein could be processed, assembled, and presented to the surface of the pEGFP-N1-VP1/PK15 cell membrane by SLA-I molecules. Therefore, the eluted peptides should be potential CTL epitopes restricted by SLA-I molecules, as previously reported [54,55]. This assay also proves that the constructed pEGFP-N1-VP1/PK15 cells can be used for screening and studying the naturally presenting CTL epitopes in vivo.

## 5. Conclusions

In conclusion, we constructed a cell line that can stably express the transfected *VP1* recombinant plasmids in PK15 based on a transient expression vector. This cell line can be used to study the presentation and elution of antigenic peptides and to further identify functional CTL epitopes matched to SLA-I molecules. This research lays the groundwork for screening CTL epitopes and developing multi-epitope FMDV vaccines.

## Figures and Tables

**Table 1:** The identified peptides derived from the VP1 protein of the O serotype of FMDV, eluted from the surface of pEGFP-N1-VP1/PK15 cell lines by LC–MS/MS.

No.	Amino Acid Sequence	Observed Mass (Da)	*m*/*z*	Charge	Sequence Length	XCorr Sequest High Throughput ^a^
1	VGALLRTA	800.4989	400.7527	2	8	1.6470474
2	TDVSFILDR *	1205.608	402.5445	3	10	1.264194489
3	QINVLDLMQ	1073.566	537.2855	2	9	1.230585575
4	QINVLDLMQ *	1074.55	537.7767	2	9	1.215035796
5	EVAVKHEGNL *	1096.563	548.7848	2	10	1.133248448
6	VLTQKAARTLP	1197.731	399.9142	3	11	1.12214005
7	QINVLDLMQTP *^b^	1289.629	645.3178	2	11	1.096101761
8	LRTATYYFA	1105.568	553.2893	2	9	1.094873786
9	NVLDLMQTPA *	1103.529	552.2638	2	10	1.065254211
10	LTQKAARTL *	1002.594	501.8008	2	9	1.051743507
11	QINVLDLMQTP *^b^	1273.634	637.3219	2	11	1.041987658
12	PNGAPEAAL	839.4258	420.2158	2	9	1.041754007
13	KVTPKDQIN *	1043.573	522.2921	2	9	0.987775028
14	VAVKHEGNLT *	1068.568	534.7877	2	10	0.986433804
15	INVLDLMQTP	1143.608	572.3118	2	10	0.978185475
16	KDQINVLDLM *	1206.592	603.796	2	10	0.948512673
17	QINVLDLMQT *	1176.582	588.7997	2	10	0.942279458
18	VTNPRGDL *	872.4472	436.7239	2	8	0.904682457
19	RGDLQVLTQ *	1030.553	515.7751	2	9	0.901747465
20	PNGAPEAAL *	840.4098	420.7085	2	9	0.896400273
21	LTWVPNGAPE	1083.547	420.7085	2	10	0.881147504
22	QKAARTLP *	885.5152	443.2597	2	8	0.85133779
23	NLTWVPNGA	971.4945	486.2554	2	9	0.841899395
24	TPKDQINVLD *	1143.589	572.293	2	10	0.839494526
25	GDLQVLTQ	873.4676	437.2395	2	8	0.83877182
26	ENYGGETQVQ	1124.485	562.7462	2	10	0.837942123
27	QINVLDLMQ *	1075.534	538.2697	2	9	0.837528408
28	PRGDLQVLTQ	1126.621	563.814	2	10	0.83436358
29	KAARTLPTSFN	1205.664	603.3296	2	11	0.831243873
30	INVLDLMQTP	1145.576	573.2885	2	10	0.830663443
31	INVLDLMQTP *	1144.592	572.8013	2	10	0.829244375
32	LQVLTQKA *	901.5353	451.2715	2	8	0.817822039
33	KAARTLPTSF	1091.621	546.3091	2	10	0.812230647
34	IKATRVTE	917.5415	459.2744	2	8	0.80939424
35	INVLDLMQ *	946.4914	473.7455	2	8	0.807365835
36	TQVQRRQH *	1055.523	528.267	2	8	0.803292811
37	RGDLQVLTQ *	1031.537	516.2765	2	9	0.802214622

* denotes a peptide modified with deamination or oxidation; ^a^ denotes the abundance value (if the value is over 1, this indicates that the peptide has a higher abundance value); ^b^ denotes different modifications in the same peptide.

## Data Availability

The datasets analyzed are available from the corresponding author on reasonable request.

## Supplementary Materials

The following supporting information can be downloaded at: https://www.mdpi.com/article/10.3390/ani15213097/s1, Figure S1: The G418 lethal concentration curve for PK15 cells. The y-axis shows the mean number of living cells after treatment. The x-axis shows the concentration of G418. Viable cells were counted on the 7^th^ day by an EVE Automatic cell counter (NanoEnTek, Seoul, Korea); Figure S2: Comparing the sequence of the VP1 in the transfected pEGFP-N1-VP1 plasmid clone and that of the VP1 in the primary pEGFP-N1-VP1 plasmid. (A) Nucleotide sequence comparison. The top sequence is from the primary plasmid, denoted as *O-VP1*, while the lower sequence is from the transfected pEGFP-N1-VP1 plasmid clone, denoted as ZB092001076 (1-PEGFP-VP1-N1) PEGFP-N5_J_G12. The nucleotide homology rate between the two sequences is 100%. (B) Amino acid sequence comparison. The top sequence is from the primary plasmid, denoted as O-VP1, while the lower sequence is from the transfected pEGFP-N1-VP1 plasmid clone, denoted as 1-PEGFP-VP1-N1.AA. The amino acid homology rate between the two sequences is 100%; Figure S3: The representative eluted peptides detected by LC–MS/MS. (A–L), the top 12 eluted peptides had XCorr Sequest High Throughput values greater than one. Among them, the peptides shown in Figure S3B, D, E, G, I, J, and K were modified with deamination or oxidation.
