# Supplementary material for: A New Strategy to Identify Naturally Presenting SLA-I Bound Peptides Derived from the O Serotype of Foot-and-Mouth Disease Virus, by Mild Acid Elution in a VP1 Stably Expressed PK15 Cell Line
Source: Animals (Basel). 2025 Oct 24;15(21):3097. doi: 10.3390/ani15213097 (PMC12606743; doi:10.3390/ani15213097)
Supplement: Supplementary file 1 [file animals-15-03097-s001.zip › animals-3851363-supplementary.pdf]

Supplementary materials

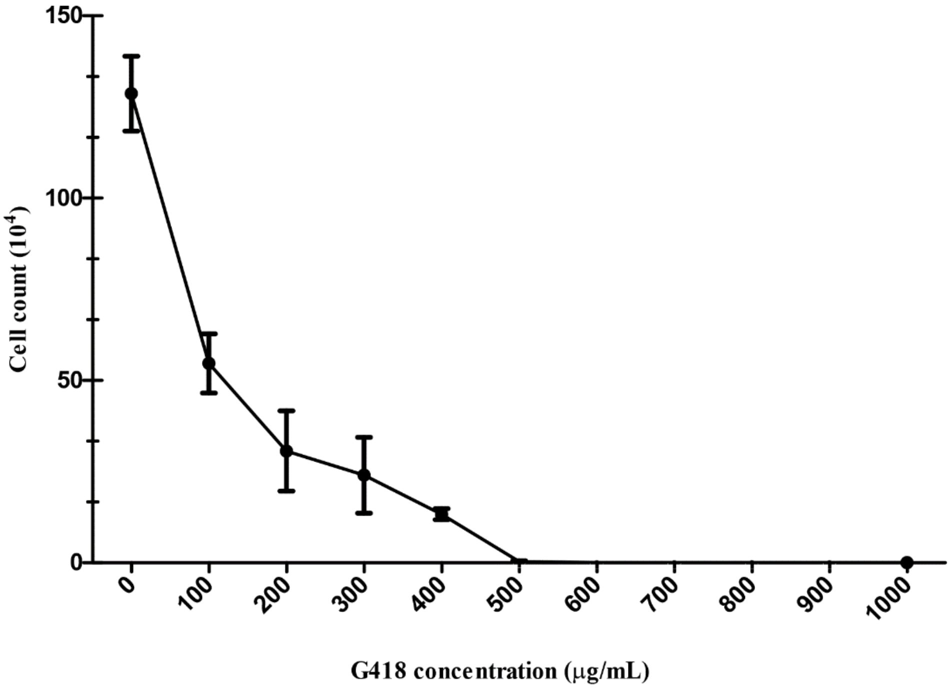

Figure S1. The G418 lethal concentration curve for PK15 cells. The y-axis shows the mean number of living cells after treatment. The x-axis shows the concentration of G418. Viable cells were counted on the 7th day by an EVE Automatic cell counter (NanoEnTek, Seoul, Korea)

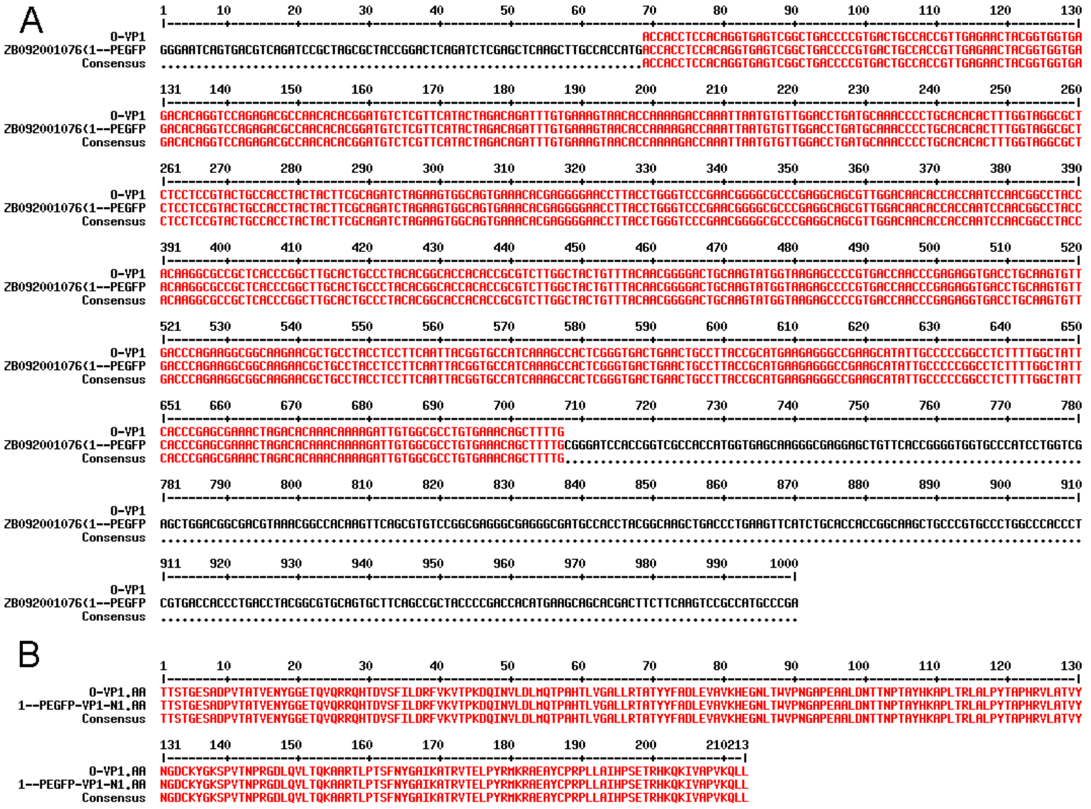

Figure S2. Comparing the sequence of the VP1 in the transfected pEGFP-N1-VP1 plasmid clone and that of the VP1 in the primary pEGFP-N1-VP1 plasmid. (**A**) Nucleotide sequence comparison. The top sequence is from the primary plasmid, denoted as O-VP1, while the lower sequence is from the transfected pEGFP-N1-VP1 plasmid clone, denoted as ZB092001076 (1-PEGFP-VP1-N1) PEGFP-N5_J_G12. The nucleotide homology rate between the two sequences is 100%. (**B**) Amino acid sequence comparison. The top sequence is from the primary plasmid, denoted as O-VP1, while the lower sequence is from the transfected pEGFP-N1-VP1 plasmid clone, denoted as 1-PEGFP-VP1-N1.AA. The amino acid homology rate between the two sequences is 100%.

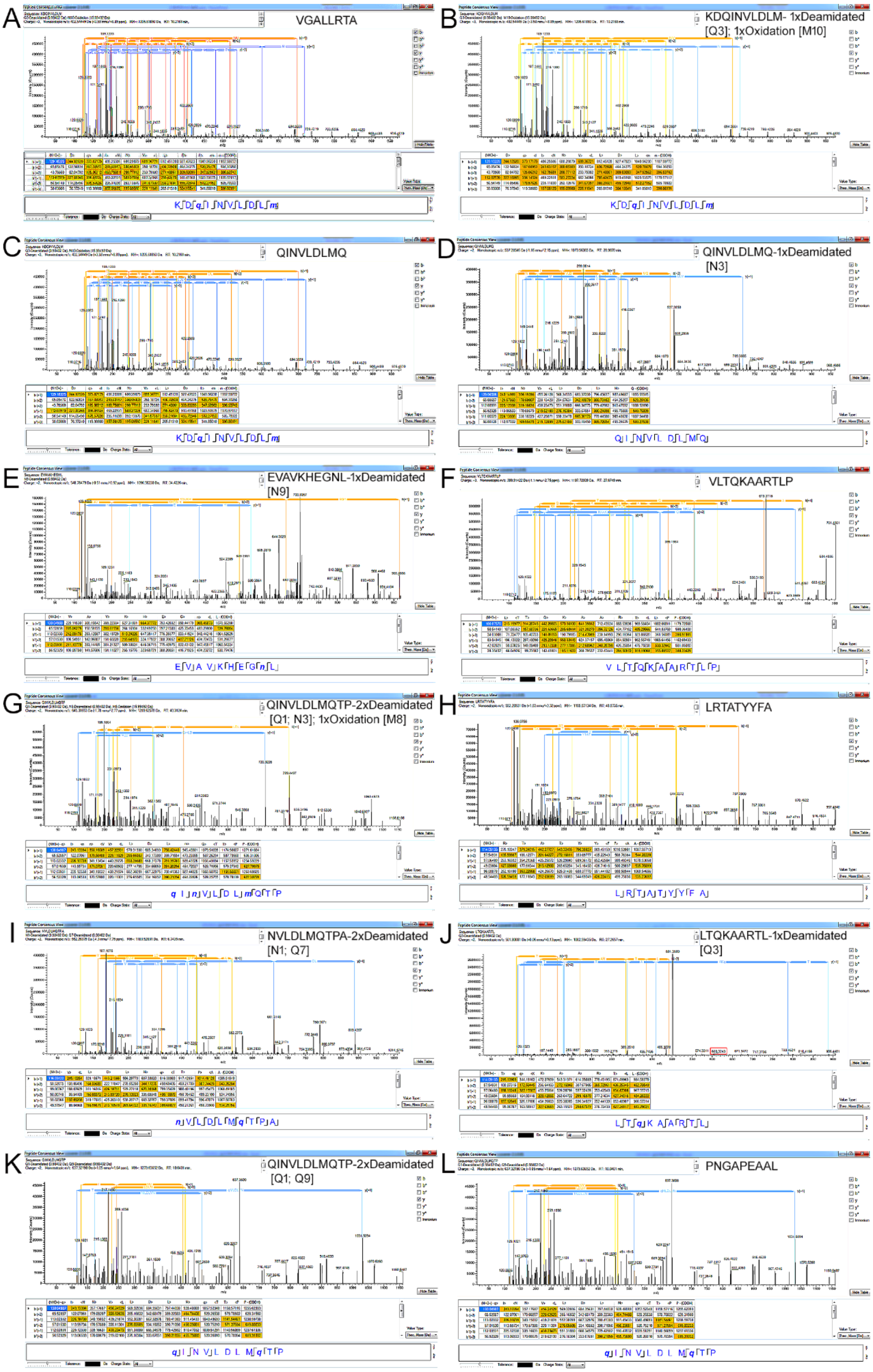

Figure S3. The representative eluted peptides detected by LC–MS/MS. (A–L), the top

12 eluted peptides had XCorr Sequest High Throughput values greater than one. Among them, the peptides shown in Figure S3B, D, E, G, I, J, and K were modified with deamination or oxidation.
